# Robot-assisted fracture fixation for pelvic fractures: a scoping review of emerging technologies

**DOI:** 10.3389/fsurg.2025.1559419

**Published:** 2025-10-14

**Authors:** Boyi Wu, Gengqi Wang, Jun Zheng

**Affiliations:** Yueyang Hospital of Integrated Traditional Chinese and Western Medicine, Shanghai University of Traditional Chinese Medicine, Shanghai, China

**Keywords:** pelvic fractures, robot-assisted fracture fixation, minimally invasive surgery, TiRobot, orthopedic trauma, surgical precision, Matta criteria

## Abstract

**Background:**

Pelvic fractures (PF) are complex injuries often requiring multidisciplinary management. Robot-assisted fracture Fixation (RAFF) systems have emerged as a promising innovation in PF treatment, offering improved precision, reduced radiation exposure, and minimally invasive techniques. This scoping review aims to synthesize the current evidence on the accuracy, safety, and efficiency of RAFF systems in managing PF, highlighting their benefits, limitations, and future potential.

**Methods:**

A scoping review was conducted adhering to PRISMA-ScR guidelines. Databases including PubMed and Web of Science were searched to identify studies evaluating RAFF systems for PF. Eligible studies involved adult patients undergoing robot-assisted interventions for PF and reported outcomes on accuracy, operative time, blood loss, and complications. Data extraction focused on study design, robotic platform, outcomes, and methodological quality assessed via MINORS and RoB-2.

**Results:**

Twelve studies were included, comprising case reports, case series, and one comparative study. RAFF systems demonstrated high accuracy in fracture reduction with reduced fluoroscopic exposure and minimal blood loss. Functional outcomes assessed by Matta criteria and Majeed scores were favorable. However, most studies were limited by small sample sizes and lack of long-term follow-up. No high-quality randomized controlled trials were identified.

**Conclusions:**

RAFF systems show significant potential in improving surgical outcomes for PF, offering enhanced precision and reduced operative risks. Nevertheless, robust, high-quality studies are needed to establish the long-term efficacy and economic viability of these systems. Standardized protocols and multicenter trials are critical for advancing the application of robotics in orthopedic trauma surgery.

## 1 Introduction

Pelvic fractures (PF) are complex, high-energy injuries often associated with life-threatening hemorrhage (1, 2). Traditional open surgery poses challenges due to excessive bleeding, leading to the adoption of minimally invasive techniques like computer-navigated percutaneous screw placement (3). Management of PF requires a multidisciplinary approach, including pelvic stabilization, angiographic embolization, and damage control strategies (2, 4). Early identification and treatment of life-threatening injuries are crucial, with a focus on controlling hemorrhage from various sources (5).

Robot-assisted fracture Fixation (RAFF) systems has emerged as a promising approach to improve surgical outcomes in pelvic fracture treatment. These systems provide benefits like precise preoperative planning, real-time 3D navigation, and minimally invasive procedures (6, 7). Studies have shown that RAFF systems achieve high accuracy in fracture reduction, with mean errors ranging from 1.3 to 3.4 mm (8). Robot-assisted percutaneous screw fixation for pelvic and acetabular fractures is more accurate, has less radiation exposure, and is more efficient than traditional methods (7, 9). However, functional outcomes appear similar between robotic and traditional methods (10).Clinical applications have shown promising results, with high rates of excellent or good outcomes based on Matta criteria (6). Integration of multiple robotic systems, such as the combination of the Starr Frame with the Da Vinci robot, can further enhance treatment options for complex cases involving sacral nerve injuries (11). As surgeons gain experience with these systems, operation times significantly decrease while maintaining accuracy (12). RAFF systems differ from traditional navigation systems primarily in their robotic manipulation capabilities. While navigation systems solely offer real-time guidance and positional feedback to surgeons for manual intervention, robotic systems additionally include mechanical manipulators capable of executing surgical actions, thus enhancing precision and reducing human variability.

The TiRobot was one of the earliest systems used for pelvic fractures, with its application in this field dating back to 2018, Jilin University First Hospital's trauma department applied TiRobot from May 2018 to April 2021 for minimally invasive percutaneous screw fixation of pelvic fractures (13). The application of robotic technology in orthopedic trauma surgery is still in its early stages, with ongoing research focusing on expanding its clinical indications and addressing limitations (14). This scoping review aimed to evaluate the role and adoption of new robotic platforms in the management of pelvic fractures, specifically assessing their application in Robot-assisted fracture Fixation and percutaneous screw fixation techniques. The objective is to identify and map the current evidence on the accuracy, safety, and efficiency of these systems compared to traditional methods, as well as to provide insights on functional outcomes and highlight future research priorities for expanding the clinical use of robotic technology in orthopedic trauma surgery.

## 2 Materials and methods

### 2.1 Search strategy and data sources

This scoping review was conducted with a systematic approach, adhering to the Preferred Reporting Items for Systematic Reviews and Meta-Analyses extension for Scoping Reviews (PRISMA-ScR) statement (15), to address the following question: “What is the current evidence supporting the performance of RAFF systems in the management of pelvic fractures?”

The initial search was conducted in PubMed to identify all emerging surgical robots currently available or undergoing testing in preclinical and clinical trials for pelvic fracture treatment. Systems identified include a real-time 3D navigation-based robot, and hybrid approaches combining mechanical tools such as the Starr Frame with robotic platforms like the Da Vinci robot and an autonomous reduction robot, while dual-robotic systems like the TiRobot and Artis Zeego also showed promise. Robotic platforms primarily focused on precise screw placement, improved accuracy in fracture reduction, and reduced surgical risks and radiation exposure, though existing multiport platforms such as the Intuitive Surgical Da Vinci robot series were excluded from the review.

In accordance with the PCC format, the following criteria were applied in selecting studies for this scoping review on RAFF systems in managing pelvic fractures:

Population (P): Adult patients (>18 years) diagnosed with pelvic fractures, requiring surgical intervention, regardless of the complexity of the fracture or underlying injury mechanism.

Concept (C): Application of Robot-assisted fracture Fixation (RAFF) systems, specifically focusing on emerging robotic technologies that enhance minimally invasive fracture reduction and percutaneous screw fixation. Systems considered include real-time 3D navigation-based robots, combinations of platforms like the Da Vinci robot, autonomous reduction robots, and dual-robotic systems such as TiRobot and Artis Zeego, while excluding traditional multiport platforms.

Context (C): Studies from clinical settings, including tertiary care and specialized trauma hospitals, where these innovative robotic systems have been implemented and evaluated for pelvic fracture treatment.

All reported outcomes across the included studies were analyzed, encompassing intraoperative, postoperative, short-term outcomes, functional results, and cost evaluations. A broad range of study designs was considered, including case reports, though review articles were excluded. Studies that contained single case reports or small series embedded within a larger series were omitted. In cases where redundancy could not be assessed due to missing registration protocol numbers or different inclusion periods, studies were flagged in the results and retained. Abstracts lacking complete case details and conference communications were excluded, with only studies published in English included in the analysis.

The literature search and selection process were conducted independently by two reviewers (B.W., J.Z.). In accordance with PRISMA guidelines, all records were compiled into a single database, duplicates were removed, and the remaining articles were screened for relevance based on titles and abstracts. Any discrepancies were resolved through discussion and consensus, and if consensus was not reached, a senior author (G.W.) was consulted to make a final determination on inclusion. Subsequently, the two reviewers conducted independent full-text reviews to confirm the eligibility of each article.

### 2.2 Data extraction and synthesis

Data extracted from the selected studies were recorded in an electronic spreadsheet. The following information was collected: first author's name, year of publication, study design, study period, type of robotic platform used, total number of patients/procedures, number of patients treated for pelvic fractures, patient demographics (including sex, age, and body mass index), fracture type and location, preoperative injury assessment, type of preoperative stabilization, surgical intervention details, number and type of robotic and assistant arms used, intraoperative outcomes, postoperative outcomes, accuracy of fracture reduction, short-term outcomes, long-term outcomes, functional recovery, learning curve of the robotic system, radiation exposure, and cost analysis.

### 2.3 Quality assessment

The quality and risk of bias in the included studies were evaluated using the MINORS (Methodological Index for Non-Randomized Studies) scoring system. In this system, each item is scored as follows: 0 if the item is not reported, 1 if the item is reported but inadequately addressed, and 2 if the item is both reported and adequately addressed. The highest possible score is 16 for non-comparative studies and 24 for comparative studies. Case reports were excluded from this quality assessment due to their inherently high risk of bias. For randomized controlled trials, the revised Cochrane risk-of-bias tool (RoB 2) was applied to ensure a comprehensive assessment (16).

## 3 Results

**Figure 1:**
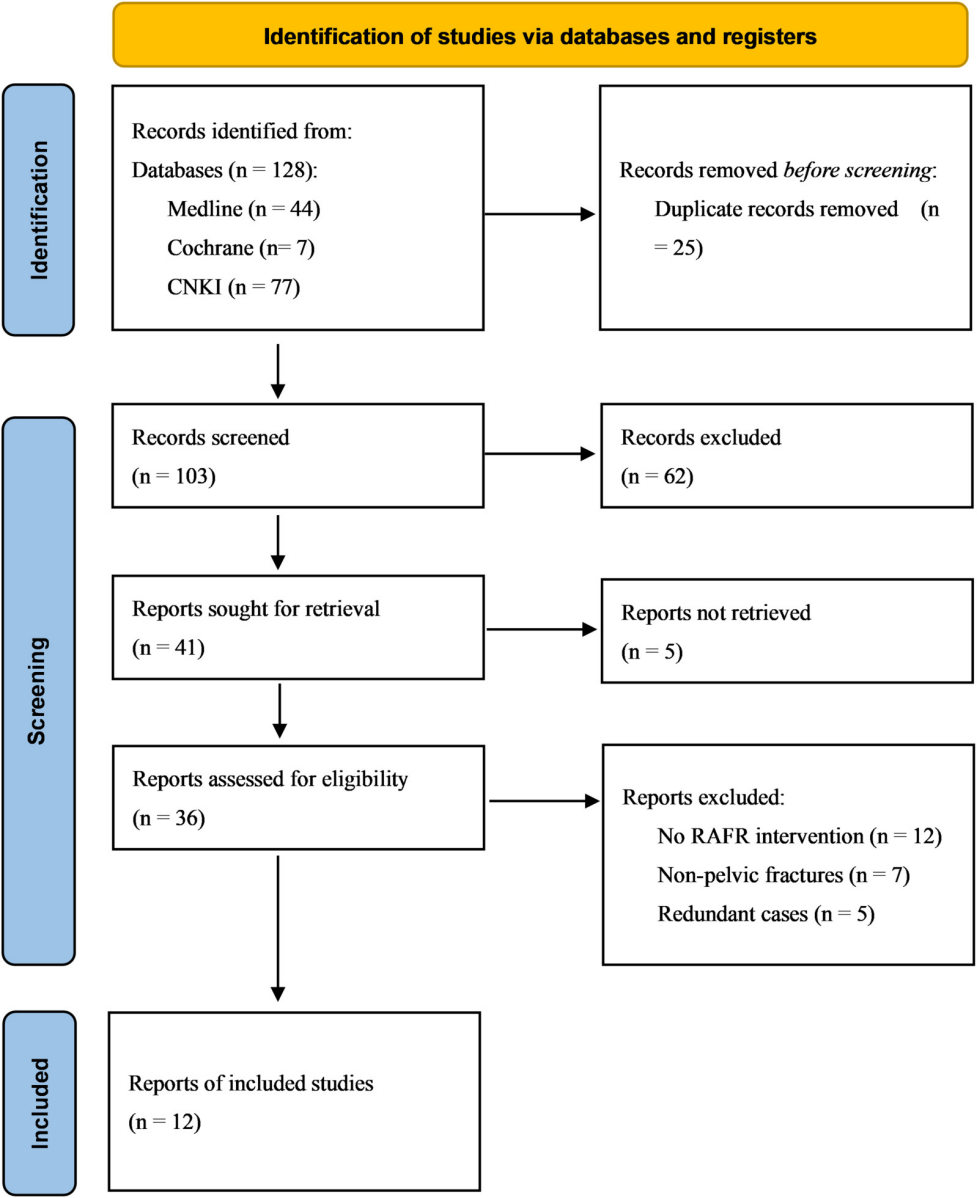
PRISMA flowchart of the literature search and selection.

The initial database search identified a total of 128 studies, of which 25 were duplicates. After screening the titles and abstracts of the 103 remaining articles, 62 were excluded due to irrelevance. Five articles could not be retrieved despite efforts, leaving 36 articles for full-text review. Among these, 24 were excluded: 12 did not involve RAFF intervention, 7 were not focused on pelvic fractures, and 5 were redundant cases. Ultimately, 12 studies met the inclusion criteria and were selected for qualitative synthesis of the literature (Figure 1).

Among the included studies, 1 was case report, 10 were case series, and 1 was comparative study. Two studies were randomized controlled trials. No randomized controlled trials (RCTs) were identified.

Overall, a total of 396 patients were included, of whom 375 underwent interventions using a RAFF system with a robotic platform. The most commonly reported intervention was percutaneous screw fixation (PSF, *n* = 191), followed by minimally invasive internal fixation (MIIF, *n* = 49), sacral screw placement (*n* = 24), and internal fixator (INFIX, *n* = 56). Additionally, 76 cases involved dual-robot-assisted or mixed interventions that were not distinctly categorized. These interventions demonstrated the versatility of robotic platforms in treating various pelvic fracture types.

The mean MINORS score for non-comparative studies ranged from 7 to 13, reflecting moderate methodological quality. The single comparative study reported a clear advantage in surgical precision and reduced fluoroscopy frequency using the TiRobot system compared to conventional methods. No studies presented a randomized design, limiting the ability to assess high-level evidence for RAFF systems.

### 3.1 Robotic platforms

The studies reviewed utilized three robotic platforms: the Rossum Robot (Beijing Jishuitan Hospital and Rossum Robot Co., Ltd., Beijing, China), TiRobot (TINAVI Medical Technologies Co., Ltd., Beijing, China), and a dual robotic system combining TiRobot with the Artis Zeego system (Siemens, Germany). The Rossum Robot featured a six-degree-of-freedom (6-DOF) manipulator, elastic traction, and mirrored path planning capabilities, enabling high precision in pelvic fracture reduction (17). TiRobot, the most commonly used system, provided sub-millimeter precision, advanced navigation, and automatic trajectory correction (21, 23, 27). The Artis Zeego system introduced real-time 3D imaging for superior surgical visualization and planning, enhancing the dual robotic system's functionality (24). The types of RAFF systems and their applications across included studies are summarized in Table 1.

**Table 1:** RAFF System adopted.

References	Robotic platform	Total cases (*n*)	RAFF systems cases (*n*)	Intervention type
Case report				
Ge et al. (6)	Autonomous reduction system	1	1	PSF
Case series (prospective and retrospective)				
Dai et al. (17)	Rossum robot, TiRobot	19	19	MIIF and INFIX
Wang et al. (18)	Intelligent robotic system	15	15	PSF and INFIX
Liao et al. (19)	Reduction robot, navigation robot	10	10	MIIF and/or INFIX
Wang et al. (20)	TiRobot	24	24	Sacral screw placement
Xia et al. (21)	TiRobot	26	26	PSF
Liu et al. (22)	TiRobot	16	16	PSF
Liu et al. (23)	TiRobot	108	108	PSF
Liu et al. (24)	TiRobot, artis Zeego	90	90	Dual-robot-assisted MIIF and PSF
Zhao et al. (25)	RAFF system	22	22	INFIX
Wu et al. (26)	RAFF system	20	20	MIIF and PSF
Comparative study				
Liu et al. (27)	TiRobot	45	24	PSF and INFIX

INFIX, internal fixator; PSF, percutaneous screw fixation; MIIF, minimally invasive internal fixation.

### 3.2 Baseline characteristics

Across the studies, the patient population included individuals aged 13–81 years, with an average age ranging from 38.5 to 56 years. Male patients predominated (55%–62%). Most fractures were classified as Tile B (68.5%–72%), with Tile C fractures comprising the remainder. Preoperative preparation involved routine CT imaging, 3D reconstructions, and skeletal traction in cases of vertical instability (6, 17, 18, 23, 26). Table 2 presents the demographic characteristics, fracture types, and comorbidities of the study population.

**Table 2:** Patient demographics, fracture classification, and comorbidities.

References	Age	Sex	Fracture type	Comorbidities
Case report				
Ge et al. (6)	56	1 M	Tile B2	NR
Case series (prospective and retrospective)				
Dai et al. (17)	51.16 (±18.47) (13.00–81.00)	10 M; 9 F	5 Tile B, 14 Tile C	NR
Wang et al. (18)	55.2 (±17.4) (30–81)	9 M; 6 F	2 Tile B, 13 Tile C	14 cases with fractures or organ injuries
Liao et al. (19)	45.5 (30–71)	6 M; 4 F	2 Tile B1, 1 Tile B2, 7 Tile B3	1 case of hemorrhagic shock, 6 cases of limb fractures, 5 rib fractures, 4 thoracolumbar fractures, 3 lung contusions, 2 bladder injuries, 3 lower extremity nerve injuries, 1 knee ligament and meniscus injury
Wang et al. (20)	49.29 (±14.48) (21–73)	10 M; 14F	13 Tile B, 11 Tile C	NR
Xia et al. (21)	42.2 (±8.6)	16 M; 10 F	6 AO/OTA 61-A2, 4 AO/OTA 61-B1, 11 AO/OTA 61-B2, 1 AO/OTA 61-B3, 3 AO/OTA 61-C1, 1 AO/OTA 61-C3, 4 AO/OTA 62-A3, 1 AO/OTA 62-B1.	20 cases with associated injuries, including 5 acetabular fractures, 8 rib fractures, 5 thoracolumbar fractures, 1 femoral fracture, 1 bilateral tibial fracture, and 2 upper limb fractures
Liu et al. (22)	38.6 (26–72)	11 M; 5 F	10 Tile B, 6 Tile C	3 rib fractures, 2 thoracolumbar fractures, 1 urethral rupture
Liu et al. (23)	41.6 (21–79)	71 M; 37 F	6 Tile A2; 45 Tile B; 57 Tile C	53 cases with associated injuries, including rib fractures, thoracolumbar fractures, and organ injuries
Liu et al. (24)	46.5 (13–78)	64 M; 26 F	33 sacroiliac fractures; 24 acetabular fractures (anterior or posterior column); 33 combined fractures	35 cases with limb fractures, 39 thoracic injuries, 8 craniocerebral injuries, 13 abdominal injuries, 28 thoracolumbar fractures
Zhao et al. (25)	50 (25–76)	15 M; 7 F	4 Tile B; 18 Tile C	19 cases with associated injuries, including rib fractures, vertebral fractures, and retroperitoneal hematoma
Wu et al. (26)	49.25 (±19.90)	11 M; 9 F	5 Tile B; 15 Tile C	17 cases with associated fractures or organ injuries
Comparative study				
Liu et al. (27)	37.4 (±6.6)	15 M; 9 F	17 Tile B; 7 Tile C	NR

NR, not reported. All the reported values are absolute, mean (range) or mean (±SD) if not specified.

### 3.3 Operative time and blood loss

Operative times varied across studies, ranging from 65 to 339 minutes, with an average of 18.9 minutes required for screw implantation in TiRobot-assisted cases. Intraoperative blood loss was minimal, averaging between 20 and 140 ml, reflecting the minimally invasive nature of the procedures (6, 19, 20, 22). Operative time, estimated blood loss, and fluoroscopy frequency are detailed in Table 3.

**Table 3:** Intraoperative data.

References	OT (min)	EBL (ml)	Fluoroscopy frequency (times)	Incision count	Incision length (cm)
Case report					
Ge et al. (6)	110	50	NR	NR	NR
Case series (prospective and retrospective)					
Dai et al. (17)	206 (203–212)^a^	100 (100–200)^a^	28 (18–55)^a^	NR	≤1
Wang et al. (18)	339.9 (±116.2) (100–525)	140.4 (±102.6) (50–400)	NR	NR	NR
Liao et al. (19)	215.5 (180–235)	110.0 (50–200)	31.8 (18–66)	NR	≈1
Wang et al. (20)	NR	NR	NR	NR	NR
Xia et al. (21)	21.3 (15–79)	20.6 (12–36)	24.6 (11–67)	NR	≈1
Liu et al. (22)	65 (50–120)^a^	35 (5–60)^a^	14 (9–27)^a^	NR	≈2
Liu et al. (23)	106 (25–240)	20.1 (5–35)	29.2 (9–63)	NR	≈2
Liu et al. (24)	18.92 (7.5–33.0)	20 (5–200)^a^	NR	NR	NR
Zhao et al. (25)	220 (120–360)	159 (50–600)	NR	NR	NR
Wu et al. (26)	206 (200.75–211.5)^b^	100 (62.5–200)^b^	29.5 (18.5–58.75)^b^	NR	NR
Comparative study					
Liu et al. (27)	65.4 (50–120)	35.0 (±7.2)	29.2 (±7.6)	5–8 per patient	≈2

OT, operative time; EBL, estimated blood loss; NR, not reported. All the reported values are absolute, mean (range) or mean (±SD) if not specified.

a Median (range).

b Median (IQR).

### 3.4 Fluoroscopic exposure and incision details

The number of fluoroscopic exposures averaged 14–32 instances per procedure, significantly lower than traditional methods. Incisions were typically less than 2 cm in length, with a mean of 1.5 cm, underscoring the minimally invasive approach (18, 20, 24, 27).

### 3.5 Postoperative outcomes and follow-up

Follow-up durations ranged from 3 to 17 months (6, 17, 19, 21). Fracture healing times averaged 3.5 months (17, 19, 27), and postoperative functional outcomes were excellent, with Majeed scores ranging from 86.7 to 89.4 (17, 22, 23). Reduction quality, assessed using Matta criteria, achieved excellent-to-good rates of 84.21%–95.8% (17, 22, 23, 26), demonstrating the effectiveness of robotic assistance in achieving anatomical alignment (17, 19, 25). Follow-up duration, healing time, and functional scores are provided in Table 4.

**Table 4:** Postoperative data.

References	FU (month)	FHT (month)	Majeed score	Matta's criteria excellent and good rate
Case report				
Ge et al. (6)	3	NR	95	100%
Case series (prospective and retrospective)				
Dai et al. (17)	17.00 (12.75–20.00)^a^	3.55 (3.35–4.18)^a^	86.00 (±6.65) (74.00–98.00)	84.21%
Wang et al. (18)	NR	NR	NR	86.7%
Liao et al. (19)	16 (13–18)	2.83 (2.53–3.22)	72.7 (70–92)	90%
Wang et al. (20)	6.00 (±3.28) (3–13)	NR	84.37 (±8.38)	95.8%
Xia et al. (21)	13.3 (7–24)	NR	80.5 (53–92)	92.3%
Liu et al. (22)	4.2 (3–6)	NR	86.7	NR
Liu et al. (23)	4.2 (3–6)	NR	89.4	NR
Liu et al. (24)	NR	NR	NR	NR
Zhao et al. (25)	NR	NR	NR	95.5%
Wu et al. (26)	NR	NR	NR	85%
Comparative study				
Liu et al. (27)	5.4 (4–12)	4.3 (±0.7)	86.4 (±7.2)	NR

FU, follow-up; FHT, fracture healing time; NR, not reported. All the reported values are absolute, mean (range) or mean (±SD) if not specified.

a Median (range).

### 3.6 Complications

Intraoperative complications were rare, with no reports of vascular or neural injuries. Postoperative issues, such as minor screw misalignments, were promptly corrected (22, 24). Only one case of cortical perforation resulted in a switch to hybrid surgery (22).

### 3.7 Quality assessment

The methodological quality of the included studies was assessed using the MINORS criteria and RoB-2 tool. For the non-comparative studies, MINORS scores ranged from 7 to 13 out of a possible 16, indicating moderate methodological quality. The only comparative study scored 17 out of 24.

Two studies were initially labeled as randomized controlled trials; however, upon review, both lacked sufficient information on randomization procedures and blinding. These were reclassified as non-randomized comparative studies. According to the RoB-2 tool, both studies were judged to have “some concerns” regarding risk of bias due to the absence of protocol registration and limited reporting of outcome blinding.

### 3.8 Economic and operational considerations

Economic analyses were limited but suggested potential cost savings due to shorter operative times and reduced radiation exposure (22, 24). Learning curve studies indicated that surgical teams required approximately 10 cases to achieve proficiency in robotic systems (23, 27).

## 4 Discussion

The integration of robotic platforms into pelvic fracture surgery has rapidly evolved, showcasing significant advancements in precision and safety over traditional approaches. Despite this, the evidence supporting the superiority of robotic systems over manual methods remains under development, primarily due to the limited availability of large-scale, high-quality studies (6, 17–27).

This review synthesizes data from studies employing robotic platforms such as the TiRobot, Rossum Robot, and dual robotic systems combining TiRobot with the Artis Zeego system. While these technologies have been instrumental in enhancing operative precision and reducing complications, their application in pelvic fractures is still relatively nascent, with most studies published between 2016 and 2024 (6, 17–27). A recurring limitation in the literature is the prevalence of small case series or single-institution experiences, which constrains the generalizability of findings. Moreover, overlapping study populations and duplicate reporting are common, as evidenced by the frequent reuse of patient data in sequential publications (6, 17–20, 24). Such overlaps not only introduce potential selection and reporting bias but also limit the overall novelty of available evidence, as multiple studies may derive from the same institutional database. This concern underscores the need for more diverse, independent, and multicenter research efforts.

Furthermore, the strength of the evidence is limited by the predominance of retrospective case series and non-comparative designs, which inherently carry a higher risk of bias. Patient characteristics and comorbidities are often underreported, with most studies focusing on basic demographics such as age and fracture classification. Although this provides a baseline understanding, it fails to account for critical factors like the Charlson Comorbidity Index or the American Society of Anesthesiologists (ASA) score, which are pivotal for evaluating postoperative risks and outcomes (19, 22). Preoperative preparations, including 3D imaging and skeletal traction, were standardized across studies, facilitating the adoption of advanced robotic systems (18, 20).

Intraoperative outcomes are promising, with robotic platforms demonstrating reduced operative times, minimal blood loss, and low fluoroscopic exposure compared to conventional techniques. Incision lengths and fluoroscopy frequency are consistently reported as minimal, reflecting the advantages of minimally invasive procedures (21, 23, 26). However, these benefits are partially offset by the steep learning curve associated with robotic systems, as surgical teams require approximately 10–15 cases to achieve proficiency (24, 25, 27).

Postoperative outcomes highlight the effectiveness of robotic platforms in achieving high-quality reductions and functional recovery. Matta criteria reveal excellent-to-good reduction rates exceeding 90% in most studies, and Majeed scores consistently indicate favorable functional outcomes (17, 21, 24, 26). Nonetheless, the studies often lack long-term follow-up, limiting the assessment of sustained functional and structural integrity.

A significant limitation is the sparse reporting of complications, with most studies emphasizing their rarity but failing to provide detailed accounts. While robotic systems reduce the risk of vascular or neural damage, minor complications such as screw misalignment are occasionally noted (6, 18, 21). Economic analyses remain scarce, though preliminary data suggest cost savings due to shorter operative times and reduced radiation exposure (25, 27). Moreover, very few studies pre-registered protocols or reported detailed bias mitigation strategies, which further limits confidence in their findings. Future studies should adhere to standardized reporting and methodological rigor to enhance transparency and comparability.

Future research must prioritize multicenter randomized controlled trials to validate the clinical and economic benefits of robotic platforms. Given the steep learning curve and institutional concentration of existing data, future trials should also address the generalizability of findings across diverse settings and surgical teams. Additionally, the transferability of robotic skills across platforms and the role of expertise in optimizing outcomes warrant further exploration. As robotic technologies continue to evolve, integrating standardized reporting frameworks and comprehensive data collection will be crucial for advancing the field (21, 24, 27).

In addition, mechanical reduction frames such as Matta's frame, widely used for pelvic fracture reduction, should be considered separately from robotic platforms due to their purely mechanical nature without robotic manipulation.

## 5 Conclusions

RAFF systems represent a transformative advancement in the surgical management of pelvic fractures, providing improved precision, reduced radiation exposure, and minimized invasiveness compared to traditional methods. Platforms such as the TiRobot, Rossum Robot, and dual systems combining TiRobot with Artis Zeego have demonstrated feasibility and safety in achieving high-quality fracture reductions, as evidenced by favorable Matta criteria and Majeed scores. Despite these advantages, the current literature is predominantly based on small-scale studies, case series, and single-center experiences, with no high-quality randomized controlled trials available.

Functional outcomes following robotic-assisted procedures appear comparable to traditional methods, but the overall benefits of robotics—reduced blood loss, shorter fluoroscopic exposure, and enhanced accuracy—highlight their potential in improving patient recovery and safety. However, the steep learning curve and limited economic evaluations remain significant challenges.

Future research should prioritize multicenter, randomized controlled trials and standardized reporting of outcomes to validate the clinical and economic efficacy of RAFF systems. Expanding the use of robotics in orthopedic trauma surgery will require addressing these knowledge gaps and integrating advanced technologies with surgical expertise. Robust evidence will ultimately determine the long-term role of robotic platforms in transforming the management of pelvic fractures.

## References

[1] Moreno C Moore EE Rosenberger A Cleveland HC. Hemorrhage associated with major pelvic fracture: a multispecialty challenge. J Trauma Acute Care Surg. (1986) 26:987. 10.1097/00005373-198611000-00005 3783790

[2] Wong JM-L Bucknill A. Fractures of the pelvic ring. Injury. (2017) 48:795–802. 10.1016/j.injury.2013.11.021 24360668

[3] Yu T Cheng X-L Qu Y Dong R-P Kang M-Y Zhao J-W. Computer navigation-assisted minimally invasive percutaneous screw placement for pelvic fractures. WJCC. (2020) 8:2464–72. 10.12998/wjcc.v8.i12.2464 32607323 PMC7322419

[4] Biffl WL Smith WR Moore EE Gonzalez RJ Morgan SJ Hennessey T Evolution of a multidisciplinary clinical pathway for the management of unstable patients with pelvic fractures. Ann Surg. (2001) 233:843. 10.1097/00000658-200106000-00015 11407336 PMC1421328

[5] Matsushima K Love B Tadlock MD. Damage Control for Pelvic Fracture Bleeding. Cambridge: Cambridge University Press (2019). p. 335–42. 10.1017/9781108698665.038

[6] Ge Y Zhao C Wang Y Wu X. Robot-assisted autonomous reduction of a displaced pelvic fracture: a case report and brief literature review. JCM. (2022) 11:1598. 10.3390/jcm11061598 35329924 PMC8950953

[7] Wu X Wang J Sun X Zhao C. Guidance for treatment of pelvic acetabular injuries with precise minimally invasive internal fixation based on the orthopaedic surgery robot positioning system. Orthop Surg. (2019) 11:341–7. 10.1111/os.12452 31062515 PMC6595112

[8] Shi C Zhao X Wu X Zhao C Zhu G Shi S Real-Time 3D navigation-based semi-automatic surgical robotic system for pelvic fracture reduction. 2021 IEEE/RSJ International Conference on Intelligent Robots and Systems (IROS) (2021). p. 9498–503. 10.1109/IROS51168.2021.9636647

[9] Zhao CP Wang J Su Y Han W Zhou L Wang MY. Clinical research on robot-assisted percutaneous pelvic and acetabular screws surgery. J Peking Univ. Health Sci. (2017). 28416838

[10] Schuijt HJ Hundersmarck D Smeeing DPJ van der Velde D Weaver MJ. Robot-assisted fracture fixation in orthopaedic trauma surgery: a systematic review. OTA International. (2021) 4:e153. 10.1097/OI9.0000000000000153 34765903 PMC8575426

[11] Peng Y Zhang W Zhang G Wang X Zhang S Ma X Using the starr frame and Da vinci surgery system for pelvic fracture and sacral nerve injury. J Orthop Surg Res. (2019) 14:29. 10.1186/s13018-018-1040-6 30683121 PMC6347760

[12] Liu K You M Huang M Chen C Rui B Gao H Preliminary application study of dual-robotic navigated minimally invasive treatment by TiRobot and artis zeego on pelvic fractures. Chin J Reparative Reconstr Surg. (2022). 10.7507/1002-1892.202203026 PMC9379448 35979781

[13] Guang Y Baochang Q Tianhao Z. Efficacy of TiRobot-assisted minimally invasive percutaneous screw fixation for pelvic fractures. Chin J Orthop Trauma. (2022) 24:200–5. 10.3760/cma.j.cn115530-20211206-00561

[14] Zhu Z Zheng G Zhang C. Development and clinical application of robot-assisted technology in traumatic orthopedics. Chin J Reparative Reconstr Surg. (2022). 10.7507/1002-1892.202206097 PMC9379455 35979779

[15] Tricco AC Lillie E Zarin W O’Brien KK Colquhoun H Levac D PRISMA extension for scoping reviews (PRISMA-ScR): checklist and explanation. Ann Intern Med. (2018) 169:467–73. 10.7326/M18-0850 30178033

[16] Sterne JAC Savović J Page MJ Elbers RG Blencowe NS Boutron I Rob 2: a revised tool for assessing risk of bias in randomised trials. Br Med J. (2019) 366:l4898. 10.1136/bmj.l4898 31462531

[17] Dai Y Zeng Y Wu Z Zhao C Wang J Wu X. Clinical efficacy of intelligent pelvic fracture reduction robots combined with TiRobot for unstable pelvic fractures. J Capital Med Univ. (2024) 45:763–72.

[18] Wang X Wu Z Zeng Y Li C Zhou J Hong S Intelligent robot-assisted percutaneous fixation of pelvic fractures. Chin J Orthop Surg. (2024) 32:1507–10. 10.20184/j.cnki.Issn1005-8478.100512

[19] Liao J Dai Y Wu Z Zeng Y Li C Wang X Analysis of the efficacy of reduction robots combined with navigation robots in minimally invasive treatment of tile B-type pelvic fractures. Chin J Reconstr Surg. (2024) 38:954–60. 10.7507/1002-1892.202404049 PMC11335582 39175317

[20] Wang X Wu Z Zeng Y Li C Zhou J Hong S. Accuracy and clinical efficacy of robot-assisted sacroiliac screw placement for posterior pelvic ring fractures. Chin J Orthop. (2024) 37:605–8. 10.12200/j.issn.1003-0034.20240083 38910384

[21] Xia H. Liao Y. Kaesel Wang S. Mao F. Zhang D. Zhao J. Application of orthopedic robots in internal fixation of pelvic and acetabular fractures. Chin J Bone Jt Inj 2022, 37, 1284–7.

[22] Liu H Duan S Jia F Liu S Chen H Wang Y TiRobot-assisted percutaneous hollow screw fixation for unstable pelvic fractures. J Shandong Univ. (2017) 55:103–9.

[23] Liu H Duan S Zhao G Zhang Z Zhu L Wang X Clinical analysis of 108 cases of orthopedic robotic-assisted minimally invasive treatment of pelvic ring injuries. J Shandong Univ. (2019) 57:52–9.

[24] Liu K You M Huang M Chen C Rui B Gao H Preliminary application of the TiRobot combined with the artis zeego system in minimally invasive treatment of pelvic fractures. Chin J Reconstr Surg. (2022) 36:929–33. 10.7507/1002-1892.202203026 PMC9379448 35979781

[25] Zhao C Cao Q Sun X Wu X Zhu G Wang Y. Intelligent robot-assisted minimally invasive reduction system for reduction of unstable pelvic fractures. Injury. (2023) 54:604–14. 10.1016/j.injury.2022.11.001 36371315

[26] Wu Z Dai Y Zeng Y. Intelligent robot-assisted fracture reduction system for the treatment of unstable pelvic fractures. J Orthop Surg Res. (2024) 19:271. 10.1186/s13018-024-04761-5 38689343 PMC11059586

[27] Liu H Duan S Liu S Jia F Zhu L Liu M. Robot-assisted percutaneous screw placement combined with pelvic inter nal fixator for minimally invasive treatment of unstable pelvic ring f ractures. Robot Comp Surg. (2018) 14(5):e1927. 10.1002/rcs.1927 PMC6175104 29920914

